# Prediction of peptides binding to MHC class I and II alleles by temporal motif mining

**DOI:** 10.1186/1471-2105-14-S2-S13

**Published:** 2013-01-21

**Authors:** Cem Meydan, Hasan H Otu, Osman Uğur Sezerman

**Affiliations:** 1 Bioengineering Department, Sabancı University, 34956, Istanbul, Turkey; 2 Department of Medicine, BIDMC Genomics Center, Harvard Medical School, Boston, MA 02215, USA; 3 Department of Bioengineering, Istanbul Bilgi University, 34060, Istanbul, Turkey

## Abstract

**Background:**

MHC (Major Histocompatibility Complex) is a key player in the immune response of most vertebrates. The computational prediction of whether a given antigenic peptide will bind to a specific MHC allele is important in the development of vaccines for emerging pathogens, the creation of possibilities for controlling immune response, and for the applications of immunotherapy. One of the problems that make this computational prediction difficult is the detection of the binding core region in peptides, coupled with the presence of bulges and loops causing variations in the total sequence length. Most machine learning methods require the sequences to be of the same length to successfully discover the binding motifs, ignoring the length variance in both motif mining and prediction steps. In order to overcome this limitation, we propose the use of time-based motif mining methods that work position-independently.

**Results:**

The prediction method was tested on a benchmark set of 28 different alleles for MHC class I and 27 different alleles for MHC class II. The obtained results are comparable to the state of the art methods for both MHC classes, surpassing the published results for some alleles. The average prediction AUC values are 0.897 for class I, and 0.858 for class II.

**Conclusions:**

Temporal motif mining using partial periodic patterns can capture information about the sequences well enough to predict the binding of the peptides and is comparable to state of the art methods in the literature. Unlike neural networks or matrix based predictors, our proposed method does not depend on peptide length and can work with both short and long fragments. This advantage allows better use of the available training data and the prediction of peptides of uncommon lengths.

## Background

MHC (Major Histocompatibility Complex) is a large gene family with an important role in the immune system, autoimmunity, and reproduction. MHC molecules assume roles in the presentation of peptides, including self and non-self (antigenic) on their surface to T-cells. T-cells recognize antigenic peptides and trigger a cascade of events which leads to the destruction of pathogens and infected cells. Since MHCs have a key role in immune response, they are critical in many diseases, and can be used for controlling specific immunological processes by creating peptides to bind to specific MHC alleles. This binding affinity to specific peptides may be exploited for creating peptide vaccines for emerging pathogens [1], suppressing specific alleles in organ transplants [2,3], and many other possible areas in immunotherapy.

MHC class I molecules bind short peptides, whose N- and C-terminal ends are anchored into the pockets located at the ends of the peptide binding groove [4]. While the majority of the peptides are of length 9, longer peptides can be accommodated by the bulging of their central portion [5,6], resulting in binding peptides of length 8 to 15 [7]. Peptides binding to class II proteins are not constrained in size [8,9] and can vary from 11 to 30 amino acids long [10]. The peptide binding groove in the MHC class II molecules is open at both ends, which enables binding of peptides with relatively longer length. Though the "core" nine residues long segment contributes the most to the recognition of the peptide, the flanking regions are also important for the specificity of the peptide to the class II allele [11,12]. MHC molecules bind peptides with high promiscuity; it is estimated that each HLA (human leukocyte antigen system) protein can bind between 1000 and 10,000 peptides for class I allotypes [13] and more than 2000 peptides for class II allotypes [14]. Thus, the large number of possible structures makes it unfeasible to find peptides that will bind to a specific allele using solely an experimental approach.

Computational methods for prediction of the binding affinity of a peptide to an MHC allele are based on three main artificial learning systems: statistical, structural, and neural methods [13,15,16]. The combination of these models is also common [17]. Computational approaches available for predicting MHC binding peptides from amino acid sequences include: (i) Motif-based methods such as methods that use a position weight matrix (PWM) to model a gapless multiple sequence alignment of MHC binding peptides [4-8], and a statistical approach based on Hidden Markov Models (HMMs) [9,10]; (ii) Machine learning methods based on Artificial Neural Networks (ANN) [6,11-13] and Support Vector Machines (SVMs) [14-17]; (iii) Semi-supervised machine learning methods [18,19]. Existing methods are reviewed in detail in [18,19].

The formation of bulges and loops may allow peptides that are shorter or longer than 9 amino acids to bind to class I alleles. This length variance shifts the positions of amino acids in anchor locations, causing position-specific scoring matrices or other position-dependent methods to fail. Most existing methods enforce a length constraint of 9 peptides for class I prediction. ANN, quantitative matrices and similar methods require the peptides to be of the same length, with appropriate peptides aligned in the same location. Peptides of different lengths are either ignored or grouped into separate datasets by their length. This step may not always be feasible if the data is limited, especially for short and variable peptides.

Unlike MHC class I prediction methods, most of the MHC class II prediction methods can utilize peptides of variable length. However, the prediction strategy requires the determination of the core 9-mer region of the peptide. This core segment is assumed to be fixed-length and the possibility of longer binding core sequences is disregarded. Although peptides bind to MHC class II alleles mostly by the anchor residues, the interactions of the flanking regions may be important for specificity and therefore have to be taken into account [20].

In order to overcome these obstacles, we suggest a method using partial periodic pattern mining, which does not require the peptides to be of same length or the anchor positions to be specific. We propose a novel method for extracting the motifs on peptides with variable lengths by finding partial motifs in sequence data. Our method, called MHC-PPM, may capture aforementioned variations in peptides, without filtering or pre-processing the shorter/longer peptides or treating them as separate datasets. Additionally, the information in the flanking regions of the core 9-mers is taken into account without any information loss that may have arisen due to length constraints.

## Methods

### Dataset

We used 28 different alleles from the Immune Epitope Database (IEDB) benchmark dataset by Peters et al. [21,22] for MHC class I prediction (total of 36,829 peptides). For MHC class II, we used two benchmark sets from Wang et al., 16 alleles containing 10,017 peptides [19] (referred to as Wang2008), and 26 alleles containing 44,541 peptides [23] (referred to as Wang2010). Wang2010 contains data from several different human alleles, including HLA DR, DP and DQ. Wang2010 data also contains a similarity reduced subset (SR), where sequence similarity is minimized in order to reduce the overlap between cross-validation folds. In Peters and Wang2010 datasets, the same cross-validation folds are used for comparison to the benchmark values. 10-fold cross-validation was used in Wang2008 dataset.

The peptides from these alleles are assigned into positive and negative classes by the IC_50 _= 500 nM cut-off. Unlike other MHC prediction methods, no filtering was made with regard to length during the motif mining and prediction steps.

### Motif mining

#### i-) Apriori method

Our motif mining method is based on the apriori algorithm used in frequent association rule discovery [24]. An "itemset" is defined as a set of items or events that co-occur frequently. The Apriori algorithm uses the principle that all subsets of a frequent itemset must also be frequent. Accordingly, the algorithm has a bottom-up approach where the shorter frequent itemsets are extended to create longer candidates, which are then filtered by frequency of occurrence [24-26]. This iterative extension process continues until no frequent itemsets of a certain length can be found.

Due to the context difference, the formal statement of the problem in the Apriori algorithm [26] is slightly modified. Let *I *= {*i*1, *i*2*,..., im*} be an alphabet of items called *events *(amino acids in our case). Let *D *be a set of sequences, where each sequence *S *is an ordered set of items such that *S *⊆ *I*. A sequence *S *contains *itemset X*, an ordered set of some items in *I*, if *X *⊆ *S*. A rule is of the form, where *X *⊂ *I*, *Y *⊂ *I*. With temporal information, {*X*→*Y*} also implies that the events in X occur before Y in a sequence S containing the rule.

The ratio of the sequences containing the association rule to all of the sequences is called the support of the rule. The ratio of the sequences containing a new rule created by the combination of two rules to the sequences containing the previous rule is called the confidence. That is,

$$Conf\left(X\to Y\right)=\frac{Support\left(X\cup Y\right)}{Support\left(X\right)}$$

Our motif mining method (MHC-PPM) is similar to temporal event mining in time-related databases [27]. In general, the partial periodic pattern mining algorithms for time series data will attempt to find frequently co-occurring events, or causality relationships between them. These methods try to capture the patterns which occur in an order which is not necessarily a consecutive one. In the domain of protein motifs, the amino acids become the "events" and the causality/future prediction aspects become the motifs that are sought [28].

In the proposed approach, each sequence is taken as a separate time series, with many parallel events occurring at the same time, with each event related only to the sequence upon which it is found. In these time series, if an event happens frequently after another one within a given time window, this frequent occurrenceis considered an episode of events, a motif. To exploit the apriori principle for performance, the motifs begin from length 1. A longer motif including a specific amino acid will have support less than or equal to the support of that amino acid. Hence, if an amino acid is infrequent, any motif that includes that amino acid will also be infrequent. Thus, iteratively L_N _(frequent itemset of size N) is created from filtering of C_N_, candidate itemset of size N by *C_N _*= *L*_*N*-1_→*L*_1_.

First L_1, _the frequent itemsets of size 1 (i.e. amino acids) are found. The first step is straightforward: only the amino acids within the sequences are counted, and if an amino acid's frequency (support of the rule) is below the given threshold, the amino acid is filtered out.

Then the candidate set of size 2, C_2 _is created from the amino acids by L_1_→L_1_, that is, the combination of any two frequent itemsets of size 1. For example, if all of the 20 amino acids were frequent, we would have 400 candidate rules at C_2 _for the given parameters. Those candidate rules would then be filtered according to the preset minimum support values, yielding L_2_. Only a handful of those 400 rules would be frequent in the data. An example rule of size 2 would be {L→V}, which represents Leucine followed by Valine in a window specified by parameters. The support of this candidate rule will be the ratio of occurrence of L→V to all of the sequences, and the confidence of the rule would be the ratio of occurrence of L→V to all of the sequences that contain "L" at some point. In other words, confidence would be the conditional probability of seeing Valine in the window, given that we observed a Leucine.

To account for the position variations in the alleles, a specific window should be defined. If an amino acid X is followed by Y after at least *MinS *and at most *MaxS *positions, then the rule {X → Y} is present in that sequence. If these amino acids co-occur within this window by this specific order at least *minimum support *times, then it is considered frequent.

In the motif mining context, the frequent rules are not simply association rules as in a shopping basket analysis; items also have a temporal value, which is used for relations such as "before" and "after" ("simultaneously" is not used in protein motifs since at each time point, that is a specific position in the sequence, only one amino acid can occur). The episode A→B then becomes, "whenever the events in the rule A occur in a given sequence, event B is likely to occur within *n *to *m *positions after A, with P(A → B) as *p (support) *and P(B | A) as *c (confidence)*".

**Figure 1:**
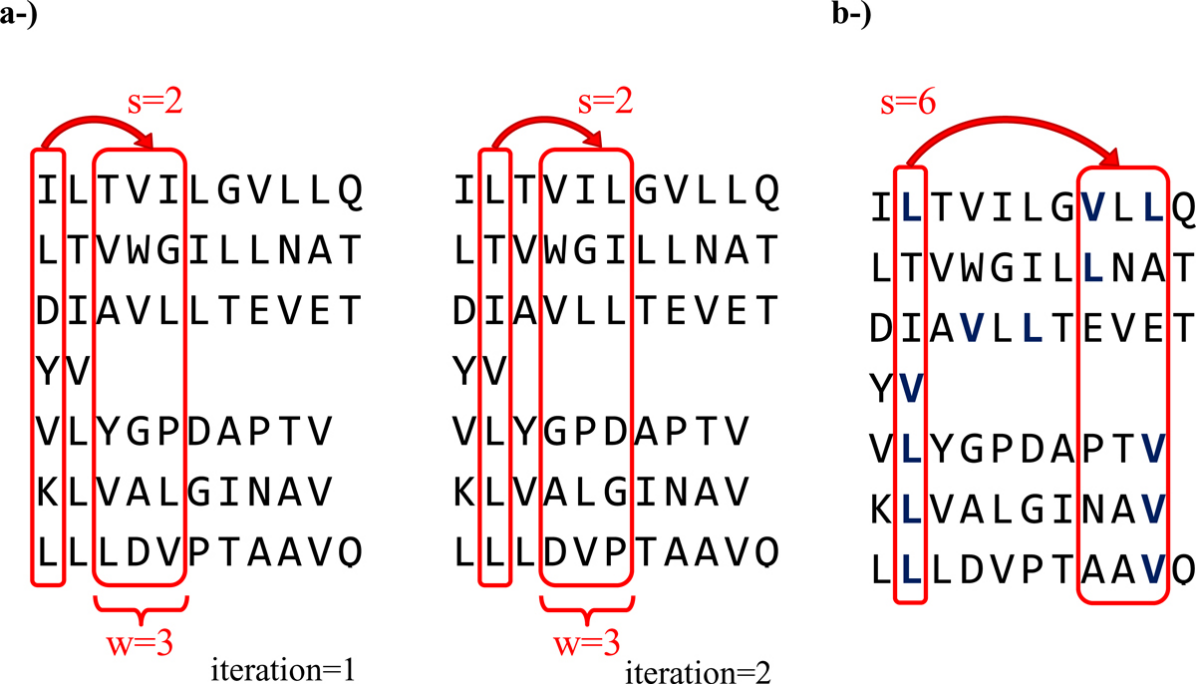
**An example of the temporal rule mining process**. **a-) **Schematic representation of the sliding windows approach on a sample set of sequences binding to MHC class I allele HLA A*0201. The windows are shifted with the given s and w values until all sequences are covered. The resulting rules are then filtered by their support. This process is repeated for all s values from -8 to -1 and 1 to 8. **b-) **Representation of the L→V rule captured with the parameters s = 6 and w = 3. For HLA A*0201, Leucine in 2^nd ^position and Valine around 9^th ^position is a well-known binding motif [40]. Although this motif is present in 6 of the 7 sequences (support of 0.86), it is unlikely to be captured by position specific methods due to length variance and positional shifts.

**Figure 2:**
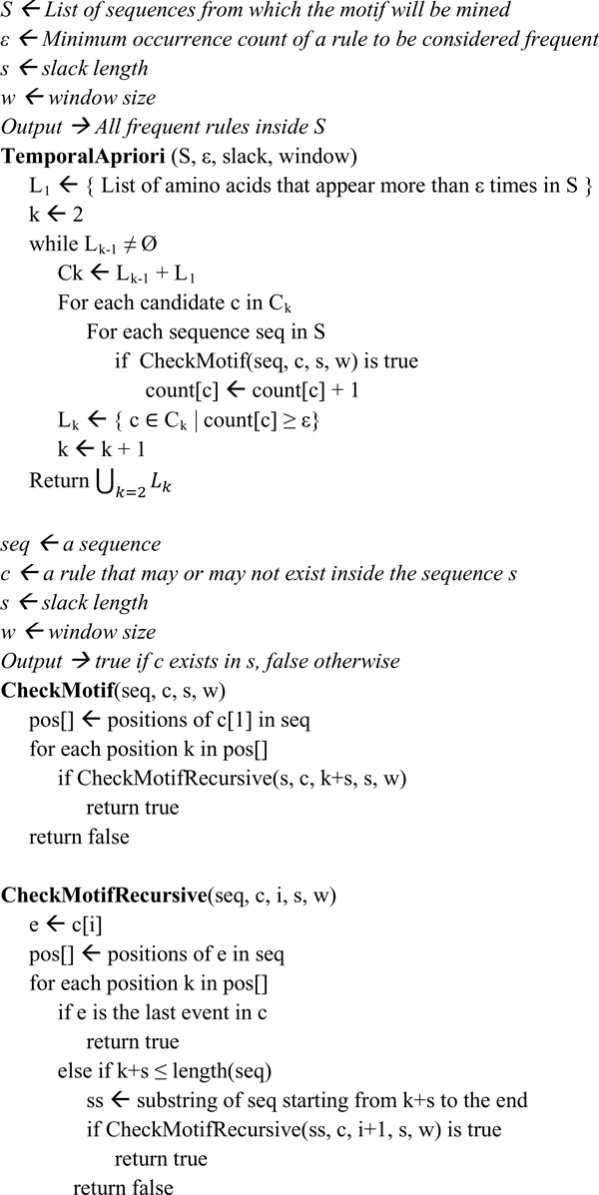
**Pseudocode of the temporal apriori motif mining algorithm**.

There are two parameters, the slack length (*s*), which is the length after an event within which we do not look for a rule, and the window size (*w*), in which the consequent event may occur. Thus, *MinS *= *s *and *MaxS *= *s *+ *w *- 1, and the rule is given as A→B (p, c) for parameters (s, w). An example motif mining step is given in Figure 1 and Additional File 1 the pseudocode of the algorithm is given in Figure 2.

In our simulations, we used a window size of 1 to 3 and slack length of -8 to 8, producing different rulesets. Negative slack values are taken by reversing the input sequences and applying the algorithm with the absolute value of the slack.

For *s *= 1 and *s *= -1, the rules that consist of consecutive/nearby amino acids were mined whereas for the larger values of s, the motifs consisting of amino acids at separate ends of the peptide were found. Since the anchor positions of MHC motifs may be different, different slack lengths are needed to mine them all.

#### ii-) Position dependent 1-rules

With the addition of position information, single amino acids can be employed as rules for anchors. When mining 1-rules, the position information is kept along with the window size. Thus, an example rule with window size of 2 may be {L, between positions 3-4 (support: p, confidence: c)}. This rule will be counted as present in a peptide which includes a Leucine between the positions 3 and 4.

#### iii-) Recursive rule mining on training set

In the rule mining process, the rules are mined for different slack lengths and finally 1-rules are added to the collection of rules. Following the rule mining process, all of the peptides in the training set are scored by the rules according to the Support-based prediction described below. After scoring every peptide in the training data, any peptide scoring below a predefined threshold is separated. Those separated peptides that are not sufficiently explained by the motifs are fed into the motif mining recursively.

**Figure 3:**
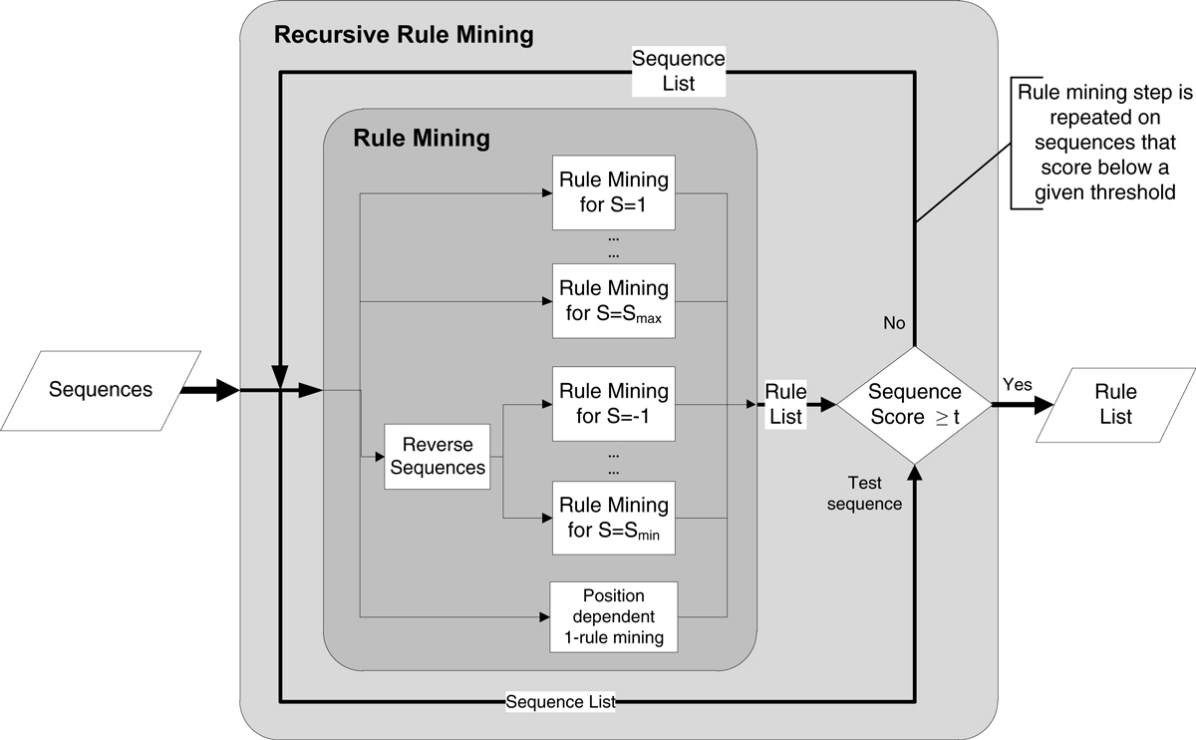
**Overall flow of the recursive rule mining step**.

This process can be thought of as mining rules for different clusters of sequences; the first iteration will try to capture the motifs for the cluster with the most sequences. After that, sequences that scored poorly will be used in motif mining again in the second iteration, and since the data is only a subset of the previous iteration, the limit for reaching minimum support will be lower. This process is repeated until the number of peptides that score lower than the threshold is below a predefined limit, until no more improvement can be gained by dividing the dataset or until a hard limit on iteration is reached. The supports for the newly mined rules are updated to reflect the support in all of the data, not the subset. An overall view of the recursive rule mining steps are given in Figure 3.

The recursive rule mining has advantages compared to setting the minimum support and confidence threshold to lower values and mining the rules in one pass. If the rules are mined in one pass with a very low support threshold, a greater number of rules will be found. Unless those rules are significant, the signal-to-noise ratio will decrease. By using a greater initial support value and progressively decreasing it on only a subset of data, the number of possible rules is reduced; if the first pass can capture motifs that are present in 70% of all sequences, we will only mine rules for explaining the remaining 30%, not the entire dataset. Hence, we end up with a lower number of more significant rules that explain the majority of the data.

### Prediction

Before prediction, rules from both the binding and non-binding sequences are mined separately. During classification of an unknown peptide, the peptide is scored independently by both the binding and non-binding rules. The simplest classification method is the direct comparison of the scores for binding/non-binding rules. To calculate the scores, the support values of the rules that occur in the given peptide are summed for both classes. The peptide is predicted to belong to the class with a higher score. This naïve approach is called Support-based prediction and only used during the recursive rule mining step.

The presence of one significant negative motif can turn an otherwise strongly binding peptide to a non-binding one. For example, in the allele H-2Kd, charged or bulky amino acids inhibit binding when they are present at the 5^th ^position, even though the binding motif may also be present [29]. In a naïve prediction method, if the binding motifs are strong enough, the large number of binder rules will overpower the single negative motif, causing a false positive. Consequently, there is need for a way to predict these enhancing/inhibiting effects of the rules. Non-linear classification methods that intrinsically find the discriminant function on the feature space would fare better in such data.

For SVR-based prediction, motif mining is employed on the training data as described above. Using the motifs, a dataset is built by creating a binary matrix, where each row is a peptide and each column (feature) represents a motif. A cell has the value 1 if the peptide corresponding to that row includes the motif, otherwise 0. As additional columns, the sums of support and confidence scores for both the positive and negative classes are given. This data matrix is built for both the training and the test sets. Then, an SVR is trained on the training set, and the binding affinities of the peptides in the test set are predicted by the support vectors. The resulting binding affinity values can be converted into a binary class using an IC_50 _threshold where a binary class is required, such as feature selection methods or AUC calculation.

Since the training set is used in all of the rule mining, SVR training and parameter optimization steps, the prediction of the test set does not include any bias and represents the actual predictive performance of MHC-PPM.

**Figure 4:**
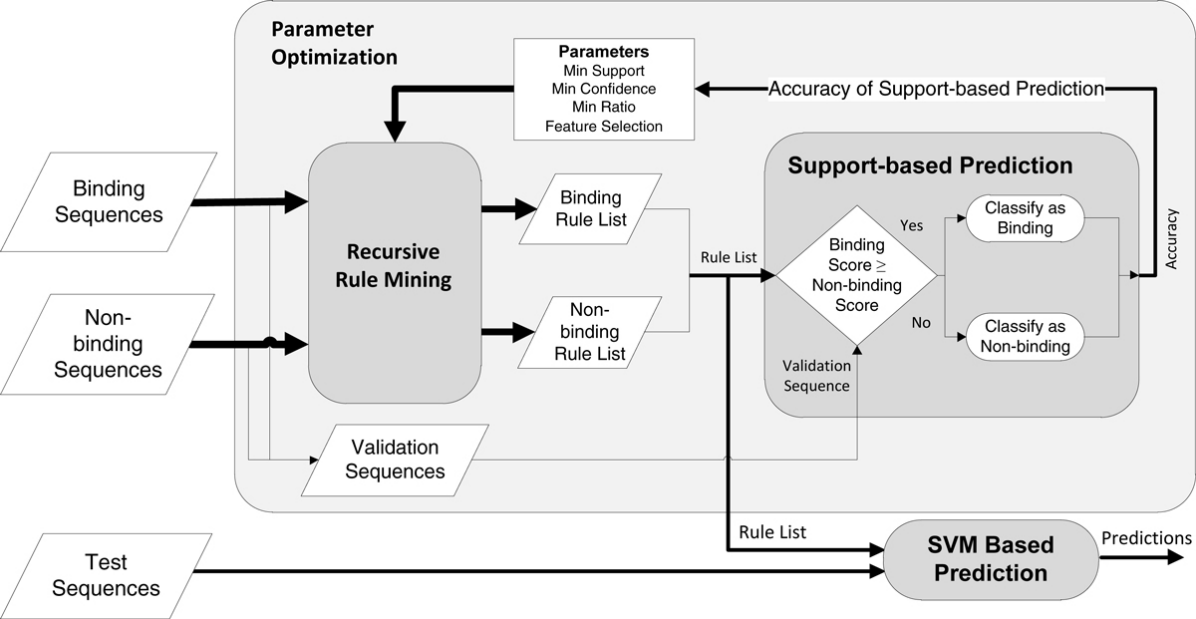
**Overview of the experimentation process**.

The overall view of the prediction workflow can be seen in Figure 4.

## Results and conclusions

### MHC class I

The prediction results of the proposed method are given in Table 1 along with the benchmark results for comparison [22]. The given values represent the area under the ROC curve (AUC) for the 5-fold cross validation using the same fold splits in the benchmark set.

**Table 1:** Results of MHC-PPM in class I predictions in Peters dataset [22].

	Allele	# Peptides		ANN	ARB	SMM	MHC-PPM
		9	10				
9-mers + 10-mers	HLA-A*0201	3089	1316	-	0.919	**0.939**	0.931
	HLA-A*0202	1447	1056	-	0.851	**0.879**	0.871
	HLA-A*0203	1443	1055	-	0.838	0.878	**0.882**
	HLA-A*0301	2094	1082	-	0.883	**0.915**	0.911
	HLA-A*0206	1437	1054	-	0.849	**0.890**	0.885
	HLA-A*1101	1985	1093	-	0.897	0.932	**0.937**
	HLA-A*2402	197	78	-	0.722	0.809	**0.833**
	HLA-A*3101	1869	1057	-	0.881	**0.903**	0.878
	HLA-A*3301	1140	1055	-	0.866	**0.888**	0.863
	HLA-A*6801	1141	1055	-	0.827	**0.874**	0.864
	HLA-B*0702	1262	205	-	0.925	0.952	**0.954**
	HLA-B*3501	736	177	-	0.833	**0.886**	0.866
	HLA-B*5101	244	177	-	0.782	0.875	**0.886**
	HLA-B*5301	254	177	-	0.758	**0.854**	0.847

9-mers	HLA-A*0101	1157	-	**0.982**	0.964	0.980	0.963
	HLA-A*2601	672	-	**0.956**	0.907	0.931	0.901
	HLA-A*2902	160	-	**0.935**	0.755	0.911	0.907
	HLA-A*6802	1434	-	**0.899**	0.865	0.898	0.867
	HLA-B*0801	708	-	**0.955**	0.936	0.943	0.926
	HLA-B*1501	978	-	0.941	0.900	**0.952**	0.922
	HLA-B*1801	118	-	0.838	0.573	0.853	**0.906**
	HLA-B*2705	969	-	0.938	0.915	**0.940**	0.938
	HLA-B*4002	118	-	0.754	0.541	0.842	**0.891**
	HLA-B*4402	119	-	0.778	0.533	0.740	**0.891**
	HLA-B*4403	119	-	0.763	0.461	0.770	**0.847**
	HLA-B*5401	255	-	0.903	0.847	**0.921**	0.883
	HLA-B*5701	59	-	0.826	0.428	0.871	**0.929**
	HLA-B*5801	988	-	0.961	0.889	**0.964**	0.944

	**Average (All)**			0.888	0.798	0.893	**0.897**
	**Average (9mers)**			0.888	0.751	0.894	**0.908**
	**Weighted Avg**			**0.932**	0.872	0.910	0.901

The best-performing method for each allele is underlined. The given AUC values for ARB and SMM are the weighted averages of the AUC values for 9-mers and 10-mers based on the given peptide counts for a specific allele. The alleles in the bottom part of the table were only trained & tested in 9-mers and are directly comparable.

Note that for some alleles (given in the top part of Table 1) the AUC values between the methods are not directly comparable because filtering of the data differs based on the prediction method in use. ANN [30] use only the 9-mers, the peptides of other lengths are filtered out. SMM uses 9-mers and 10-mers, but trained and tested independently (i.e. 9-mers belonging to an allele and 10-mers belonging to the same allele are taken as separate sets and are fed to different predictors). As stated, our method uses peptides of all lengths in the same classifier, without any filtering or separation. For the comparison in Table 1 the weighted average of AUC values using the 9-mer and 10-mer peptide counts are given for SMM [31] and ARB [32]. The results are directly comparable for alleles with only 9-mers.

Although superior in 9-mers, the main limitation of ANN is the need for the peptides to be of fixed length. The same constraint is also present in SMM and is overcome by using separate datasets for 9-mers and 10-mers. The main advantage of MHC-PPM is giving comparable and superior results to other methods without enforcing any constraints on peptide length. This flexible approach allows the use of information from all of the available data. Peptides that do not have enough representation in the dataset to train a separate classifier (e.g. 8 or 11 amino acids long) can still be predicted using the data from the 9 and 10-mers.

### MHC class II

Results for Wang2008 [19] and Wang2010 [23] datasets are given in Table 2 and Table 3 respectively. Each method in the Table 3 has results for both all of the dataset (ALL) and a similarity-reduced version of the dataset (SR), used to decrease the sequence similarity between data folds.

**Table 2:** Results of MHC-PPM in class II predictions in Wang2008 dataset [19].

Allele	#	RANKPEP	ARB	PROPRED	SMM-align	MHCMIR	MHC-PPM
HLA-DRB1*0101	3882	0.700	0.760	0.740	0.770	0.810	**0.878**
HLA-DRB1*0301	502	0.670	0.660	0.650	0.690	0.640	**0.712**
HLA-DRB1*0401	512	0.630	0.670	0.690	0.680	**0.730**	0.666
HLA-DRB1*0404	449	0.660	0.720	0.790	0.750	0.730	**0.792**
HLA-DRB1*0405	457	0.620	0.670	**0.750**	0.690	0.730	0.734
HLA-DRB1*0701	505	0.580	0.690	0.780	0.780	0.830	**0.893**
HLA-DRB1*0802	245	-	0.740	0.770	0.750	0.740	**0.827**
HLA-DRB1*0901	412	0.610	0.620	-	0.660	0.620	**0.666**
HLA-DRB1*1101	520	0.700	0.730	0.800	0.810	0.810	**0.817**
HLA-DRB1*1302	289	0.520	**0.790**	0.580	0.690	0.720	0.679
HLA-DRB1*1501	520	0.620	0.700	0.720	0.740	0.730	**0.759**
HLA-DRB3*0101	420	-	0.590	-	0.680	-	**0.712**
HLA-DRB4*0101	245	0.650	0.740	-	0.710	0.760	**0.829**
HLA-DRB5*0101	520	0.730	0.700	0.790	0.750	0.710	**0.845**
H-2 IAb	500	0.740	**0.800**	-	0.750	0.690	0.786
H-2 IEd	39	0.830	-	-	-	-	**0.867**

Average		0.661	0.705	0.733	0.727	0.732	**0.779**
Weighted Avg		0.671	0.722	0.738	0.743	0.760	**0.780**

The (#) column gives the total number of peptides for the given allele. The best-performing method for each allele is underlined.

**Table 3:** Results of MHC-PPM in MHC class II predictions in Wang2010 dataset [23].

Allele	# Peptides		ARB		SMM-align		NN-align		MHC-PPM	
	ALL	SR	ALL	SR	ALL	SR	ALL	SR	ALL	SR
HLA-DPA1*0103-DPB1*0201	1404	603	0.823	0.745	0.921	0.767	**0.943**	0.793	0.931	0.772
HLA-DPA1*01-DPB1*0401	1337	540	0.847	0.746	0.930	0.767	**0.947**	0.802	0.935	0.751
HLA-DPA1*0201-DPB1*0101	1399	604	0.824	0.743	0.909	0.786	**0.944**	0.818	0.938	0.806
HLA-DPA1*0201-DPB1*0501	1410	586	0.859	0.709	0.923	0.728	**0.956**	0.787	0.948	0.773
HLA-DPA1*0301-DPB1*0402	1407	602	0.821	0.771	0.932	0.818	**0.949**	0.828	0.935	0.815
HLA-DQA1*0101-DQB1*0501	1739	584	0.871	0.741	0.930	0.783	0.945	0.805	**0.949**	0.754
HLA-DQA1*0102-DQB1*0602	1629	593	0.777	0.708	0.838	0.734	**0.880**	0.762	0.842	0.730
HLA-DQA1*0301-DQB1*0302	1719	596	0.748	0.637	0.807	0.663	**0.851**	0.693	0.845	0.709
HLA-DQA1*0401-DQB1*0402	1701	585	0.845	0.643	0.896	0.761	**0.922**	0.742	0.920	0.778
HLA-DQA1*0501-DQB1*0201	1658	589	0.855	0.700	0.901	0.736	**0.932**	0.777	0.919	0.766
HLA-DQA1*0501-DQB1*0301	1689	602	0.844	0.756	0.910	0.801	**0.927**	0.811	0.915	0.771
HLA-DRB1*0101	6427	3504	0.770	0.710	0.798	0.756	**0.843**	0.763	0.821	0.758
HLA-DRB1*0301	1715	1136	0.753	0.728	0.852	0.808	**0.887**	0.829	0.828	0.747
HLA-DRB1*0401	1769	1221	0.731	0.668	0.781	0.721	**0.813**	0.734	0.763	0.711
HLA-DRB1*0404	577	474	0.707	0.681	0.816	0.789	0.823	0.803	**0.885**	0.717
HLA-DRB1*0405	1582	1049	0.771	0.716	0.822	0.767	**0.870**	0.794	0.831	0.734
HLA-DRB1*0701	1745	1175	0.767	0.736	0.834	0.796	**0.869**	0.811	0.846	0.804
HLA-DRB1*0802	1520	1017	0.702	0.649	0.741	0.689	**0.796**	0.698	0.752	0.687
HLA-DRB1*0901	1520	1042	0.747	0.654	0.765	0.696	**0.810**	0.713	0.762	0.671
HLA-DRB1*1101	1794	1204	0.800	0.777	0.864	0.829	**0.900**	0.847	0.858	0.811
HLA-DRB1*1302	1580	1070	0.727	0.667	0.797	0.754	**0.814**	0.732	0.768	0.717
HLA-DRB1*1501	1769	1171	0.763	0.696	0.796	0.741	**0.852**	0.756	0.813	0.745
HLA-DRB3*0101	1501	987	0.709	0.678	0.819	0.780	**0.856**	0.798	0.782	0.718
HLA-DRB4*0101	1521	1011	0.785	0.747	0.816	0.762	**0.870**	0.789	0.860	0.772
HLA-DRB5*0101	1769	1198	0.760	0.697	0.832	0.776	**0.886**	0.795	0.843	0.812
H-2-Iab	660	546	0.800	0.775	0.855	0.830	**0.858**	0.847	0.824	0.807

Average			0.785	0.711	0.849	0.763	**0.882**	0.782	0.858	0.755
WeightedAverage			0.784	0.709	0.843	0.762	**0.879**	0.778	0.853	0.754

Each method contains results from all of the peptides (ALL) and the similarity reduced data (SR). The best-performing method for each allele in ALL dataset is marked by bold and the best performing method in SR dataset is underlined.

In case of class II peptides, MHC-PPM is the top performer by the average score in the Wang2008 dataset benchmark results. However, as can be seen in the Wang2010 dataset, NN-align [33] outperforms all other methods when included in the comparison. Nonetheless, even though MHC-PPM is designed only to find position independent rules, and there are no external steps for core region detection (or any information about the core region length), it still performs exceptionally well with an average AUC value of 0.858, slightly above SMM-align [31] (AUC of 0.849) and only <0.03 lower than NN-align (AUC of 0.882).

Unlike what has been observed in class I molecules, class II molecules are believed to bind only to the core 9-mer region of a peptide. Although the core region occupies the peptide binding groove, the non-bound N- and C-terminus residues that lie outside the MHC anchor residues, called peptide flanking residues (PFRs), have been shown to affect the binding affinity and stability [11,34]. NN-align and SMM-align use the length and composition of the peptide flanking residues in addition to the peptide binding core sequence. However, to keep the same length of the input throughout the data, the flanking residues are encoded in a summarized form, decreasing the information content. Due to nature of our algorithm, the differences in affinity due to the PFRs can be captured without losing any information. To test that hypothesis, we used experimental affinity values of 9 sequences which have the same core sequence and differ only in the flanking regions [11] and tried to predict the binding affinity values from the sequence (Table 4). Although available data is limited, MHC-PPM has the lowest root mean squared error (RMSE). MHC-PPM also significantly outperforms ARB, SMM-align and NN-align in correlation of the predictions with the actual affinity values.

**Table 4:** Effect of flanking peptides on the binding affinity to HLA DRB1*1501 allele.

	Experimental	ARB	NetMHCIIpan	SMM_align	NN_align	MHC-PPM
Sequence	IC50(nM)	IC50(nM)	IC50(nM)	IC50(nM)	IC50(nM)	IC50(nM)
ENPVVHFFKNIVTPR	33	21.9	10	21	8	11
VVHFFKNIVHAAA	33	21.9	9.2	52	10.7	139
VVHFFKNIVTAAA	45	21.9	9.5	20	11.5	224
VVHFFKNIVT**K**AA	35	21.9	8.1	20	10.5	142
VVHFFKNIVTA**K**A	4	21.9	8.1	20	9.8	83
VVHFFKNIVTAA**K**	5	21.9	8.9	20	11.1	263
**D**AVVHFFKNITVA	326	82.5	23.6	25	23.8	316
A**D**VVHFFKNITVA	454	82.5	23.8	25	23.9	320
AA**D**VHFFKNITVA	264	1286.7	45	30	74.4	392

RMSE		371.90	190.78	191.84	187.13	**134.35**
Pearson's Corr.		0.349	**0.728**	0.041	0.540	0.721

Experimental affinity measurements are from [11]. Predictions of other values calculated from the IEDB website[21]. MHC-PPM has the lowest root mean squared error (RMSE) and has a correlation score approximately equal to the top performing method.

## Discussions

In this study we present a position independent motif mining method representing amino acid sequences as time series data to predict peptides binding to MHC class I and class II proteins.

In class I MHC-peptide complexes, peptides have been observed to bulge out of the binding groove [5,6], shifting the peptide side chains in the binding pockets. The main shortcoming of the existing prediction methods is their dependence on fixed length motifs, even though peptides of various lengths are known to bind to class I molecules [7]. Although a separate predictor can be created by applying the same method on a dataset of peptides of a different length, there is usually not enough available data for uncommon sequence lengths. There have been methods that use random sampling of insertions and deletions to fit the peptide into the 9-length window for prediction [35], however the fixed length limitation still present in the core.

For MHC class I predictions, MHC-PPM has been shown to slightly outperform other methods on the average. However, all methods have very close scores and perform equally well. Our main advantage is the ability to use peptides of any length during both training and prediction phases. While the curated benchmark dataset contains only 9-mers and 10-mers for the given alleles, we expect MHC-PPM to fare better in a more diverse dataset.

Commonly used prediction servers give the consensus prediction of different algorithms. The addition of our predictions into a consensus-decision step with other state-of-the-art algorithms will almost certainly benefit the end-users; the overall accuracy for the 9-mers will increase, and longer peptides that would have been previously ignored (or treated as 9-mers) will also be evaluated.

On MHC class II molecules, MHC-PPM was the top performing one in Wang2008 dataset by average AUC and just below NN-align in Wang2010 dataset. Even though NN-align outperforms all other methods including ours, the difference in performance values are not as drastic. Due to the fixed size core region, length independence is not much of an issue during score calculation. On the other hand, the position independence allows the inherent detection of the core region and allows better representation of the peptide flanking residues. During the prediction of the effects of PFRs on binding affinity (Table 4), MHC-PPM resulted in the highest agreement with the experimental data, though more data is required for conclusive results.

On the subject of peptides binding to MHC class II molecules, the current view is that the peptides lie on a shallow groove with multiple contacts along the entire length of the peptide binding groove [9,36]. This view does not address the possibility of peptides bulging out from the groove. There have been studies that proposed examples of peptide bulging (i.e. core binding region longer than 9 amino acids) in class II molecules for several alleles [9,37-39]. Even though it is not known whether this is a general phenomenon for all class II alleles, it is possible that certain alleles can anchor peptides sufficiently at their N- and C-terminals to allow bulges, similar to class I molecules. If that is the case, a length insensitive method is required to correctly identify such examples, since NN-align and other methods require a fixed length core sequence.

The strength of MHC-PPM is its ability to capture length independent short motifs that are in close vicinity. Because the motif mining and prediction steps are uncoupled, the method can be used for different purposes. We have shown that the rules mined from the data can be used in conjunction with support vector machines or neural networks for non-linear prediction of any label (or quantitative value) that is correlated with the sequence motifs. However, the actual output of the algorithm is a collection of human-understandable rules and those motifs can be used as templates during sequence analysis or synthesis. Other than MHC binding predictions, the MHC-PPM method can also be applied to find motifs in gapped sequences, such as TCR recognition or receptor-ligand prediction problems. It is straightforward to extend the method to mine multiple groups of short sequence motifs (separated by relatively long distances) which co-occur frequently. We believe this approach can help uncover previously overlooked subtle sequence motifs in any large scale data.

## Competing interests

The authors declare that there are no competing interests

## Authors' contributions

CM implemented the algorithm, carried out the experiments, contributed to the design of the study and drafted the manuscript. OUS and HHO contributed to the design as well. CM and OUS analyzed the results. CM, OUS and HHO participated in the analysis and the discussion. All authors read and approved the final manuscript.

## Declarations

The publication costs for this article were funded by the corresponding author.

This article has been published as part of *BMC Bioinformatics *Volume 14 Supplement 2, 2013: Selected articles from the Eleventh Asia Pacific Bioinformatics Conference (APBC 2013): Bioinformatics. The full contents of the supplement are available online at http://www.biomedcentral.com/bmcbioinformatics/supplements/14/S2.

## Supplementary Material

Additional File 1 **The candidate and frequent itemsets of all lengths for the given example sequences in Figure **1, **for minimum support value of 0.4**. Red/bold values represent rules above the support threshold. At each step a candidate set C_k _is generated by extending the last frequent itemset L_k-1_, then the candidates are filtered according to the support values to generate the frequent itemset L_k_. This process is repeated until no frequent itemsets of a certain size can be found. Afterwards, the resulting frequent sets of different sizes (except L_1_) are merged together and filtered according to a given minimum confidence boundary. Click here for file

## References

[B1] Rosenberg SA Yang JC Schwartzentruber DJ Hwu P Marincola FM Topalian SL Restifo NP Dudley ME Schwarz SL Spiess PJ Immunologic and therapeutic evaluation of a synthetic peptide vaccine for the treatment of patients with metastatic melanoma Nat Med 1998 4 3 321 327 10.1038/nm0398-321 9500606 PMC2064864

[B2] Zavazava N Fandrich F Zhu X Freese A Behrens D Yoo-Ott KA Oral feeding of an immunodominant MHC donor-derived synthetic class I peptide prolongs graft survival of heterotopic cardiac allografts in a high-responder rat strain combination J Leukoc Biol 2000 67 6 793 800 10857851 10.1002/jlb.67.6.793

[B3] Murphy B Kim KS Buelow R Sayegh MH Hancock WW Synthetic MHC class I peptide prolongs cardiac survival and attenuates transplant arteriosclerosis in the Lewis-->Fischer 344 model of chronic allograft rejection Transplantation 1997 64 1 14 19 10.1097/00007890-199707150-00004 9233694

[B4] Natarajan K Li H Mariuzza RA Margulies DH MHC class I molecules, structure and function Rev Immunogenet 1999 1 1 32 46 11256571

[B5] Guo HC Jardetzky TS Garrett TP Lane WS Strominger JL Wiley DC Different length peptides bind to HLA-Aw68 similarly at their ends but bulge out in the middle Nature 1992 360 6402 364 366 10.1038/360364a0 1448153

[B6] Speir JA Stevens J Joly E Butcher GW Wilson IA Two different, highly exposed, bulged structures for an unusually long peptide bound to rat MHC class I RT1-Aa Immunity 2001 14 1 81 92 10.1016/S1074-7613(01)00091-7 11163232

[B7] Schumacher TN De Bruijn ML Vernie LN Kast WM Melief CJ Neefjes JJ Ploegh HL Peptide selection by MHC class I molecules Nature 1991 350 6320 703 706 10.1038/350703a0 1708852

[B8] Nelson CA Fremont DH Structural principles of MHC class II antigen presentation Rev Immunogenet 1999 1 1 47 59 11256572

[B9] Yassai M Afsari A Garlie J Gorski J C-terminal anchoring of a peptide to class II MHC via the P10 residue is compatible with a peptide bulge J Immunol 2002 168 3 1281 1285 11801666 10.4049/jimmunol.168.3.1281

[B10] Rammensee HG Friede T Stevanoviic S MHC ligands and peptide motifs: first listing Immunogenetics 1995 41 4 178 228 10.1007/BF00172063 7890324

[B11] Godkin AJ Smith KJ Willis A Tejada-Simon MV Zhang J Elliott T Hill AV Naturally processed HLA class II peptides reveal highly conserved immunogenic flanking region sequence preferences that reflect antigen processing rather than peptide-MHC interactions J Immunol 2001 166 11 6720 6727 11359828 10.4049/jimmunol.166.11.6720

[B12] Jones EY Fugger L Strominger JL Siebold C MHC class II proteins and disease: a structural perspective Nat Rev Immunol 2006 6 4 271 282 10.1038/nri1805 16557259

[B13] Brusic V Bajic VB Petrovsky N Computational methods for prediction of T-cell epitopes--a framework for modelling, testing, and applications Methods 2004 34 4 436 443 10.1016/j.ymeth.2004.06.006 15542369

[B14] Marsh SGE Parham P Barber LD The HLA factsbook 2000 San Diego: Academic Press

[B15] Schalkoff RJ Pattern recognition: statistical, structural, and neural approaches 1992 New York: J. Wiley

[B16] Firebaugh MW Artificial intelligence: a knowledge-based approach 1988 Boston: Boyd & Fraser

[B17] Trost B Bickis M Kusalik A Strength in numbers: achieving greater accuracy in MHC-I binding prediction by combining the results from multiple prediction tools Immunome Res 2007 3 5 10.1186/1745-7580-3-5 17381846 PMC1847428

[B18] Nielsen M Lund O Buus S Lundegaard C MHC class II epitope predictive algorithms Immunology 2010 130 3 319 328 10.1111/j.1365-2567.2010.03268.x 20408898 PMC2913211

[B19] Wang P Sidney J Dow C Mothe B Sette A Peters B A systematic assessment of MHC class II peptide binding predictions and evaluation of a consensus approach PLoS Comput Biol 2008 4 4 e1000048 10.1371/journal.pcbi.1000048 18389056 PMC2267221

[B20] Matsumura M Fremont DH Peterson PA Wilson IA Emerging principles for the recognition of peptide antigens by MHC class I molecules Science 1992 257 5072 927 934 10.1126/science.1323878 1323878

[B21] Peters B Sidney J Bourne P Bui HH Buus S Doh G Fleri W Kronenberg M Kubo R Lund O The immune epitope database and analysis resource: from vision to blueprint PLoS Biol 2005 3 3 e91 10.1371/journal.pbio.0030091 15760272 PMC1065705

[B22] Peters B Bui HH Frankild S Nielson M Lundegaard C Kostem E Basch D Lamberth K Harndahl M Fleri W A community resource benchmarking predictions of peptide binding to MHC-I molecules PLoS Comput Biol 2006 2 6 e65 10.1371/journal.pcbi.0020065 16789818 PMC1475712

[B23] Wang P Sidney J Kim Y Sette A Lund O Nielsen M Peters B Peptide binding predictions for HLA DR, DP and DQ molecules BMC Bioinformatics 2010 11 568 10.1186/1471-2105-11-568 21092157 PMC2998531

[B24] Agrawal R Imielinski T Swami A Mining association rules between sets of items in large databases Proceedings of the 1993 ACM SIGMOD international conference on Management of data 1993 Washington, D.C., United States: ACM

[B25] Srikant R Agrawal R Mining generalized association rules Future Generation Computer Systems 1997 13 2-3 161 180 10.1016/S0167-739X(97)00019-8

[B26] Agrawal R Srikant R Fast Algorithms for Mining Association Rules in Large Databases Proceedings of the 20th International Conference on Very Large Data Bases 1994 Morgan Kaufmann Publishers Inc

[B27] Roddick JF Spiliopoulou M A bibliography of temporal, spatial and spatio-temporal data mining research SIGKDD Explor Newsl 1999 1 1 34 38 10.1145/846170.846173

[B28] Mannila H Toivonen H Verkamo AI Discovery of frequent episodes in event sequences Data Mining and Knowledge Discovery 1997 1 3 259 289 10.1023/A:1009748302351

[B29] Mitaksov V Fremont DH Structural definition of the H-2Kd peptide-binding motif J Biol Chem 2006 281 15 10618 10625 10.1074/jbc.M510511200 16473882

[B30] Nielsen M Lundegaard C Worning P Lauemoller SL Lamberth K Buus S Brunak S Lund O Reliable prediction of T-cell epitopes using neural networks with novel sequence representations Protein Sci 2003 12 5 1007 1017 10.1110/ps.0239403 12717023 PMC2323871

[B31] Nielsen M Lundegaard C Lund O Prediction of MHC class II binding affinity using SMM-align, a novel stabilization matrix alignment method BMC Bioinformatics 2007 8 238 10.1186/1471-2105-8-238 17608956 PMC1939856

[B32] Bui HH Sidney J Peters B Sathiamurthy M Sinichi A Purton KA Mothe BR Chisari FV Watkins DI Sette A Automated generation and evaluation of specific MHC binding predictive tools: ARB matrix applications Immunogenetics 2005 57 5 304 314 10.1007/s00251-005-0798-y 15868141

[B33] Nielsen M Lund O NN-align. An artificial neural network-based alignment algorithm for MHC class II peptide binding prediction BMC Bioinformatics 2009 10 296 10.1186/1471-2105-10-296 19765293 PMC2753847

[B34] Rotzschke O Falk K Mack J Lau JM Jung G Strominger JL Conformational variants of class II MHC/peptide complexes induced by N- and C-terminal extensions of minimal peptide epitopes Proc Natl Acad Sci USA 1999 96 13 7445 7450 10.1073/pnas.96.13.7445 10377434 PMC22105

[B35] Lundegaard C Lund O Nielsen M Accurate approximation method for prediction of class I MHC affinities for peptides of length 8, 10 and 11 using prediction tools trained on 9mers Bioinformatics 2008 24 11 1397 1398 10.1093/bioinformatics/btn128 18413329

[B36] Scott CA Peterson PA Teyton L Wilson IA Crystal structures of two I-Ad-peptide complexes reveal that high affinity can be achieved without large anchor residues Immunity 1998 8 3 319 329 10.1016/S1074-7613(00)80537-3 9529149

[B37] Fleckenstein B Kalbacher H Muller CP Stoll D Halder T Jung G Wiesmuller KH New ligands binding to the human leukocyte antigen class II molecule DRB1*0101 based on the activity pattern of an undecapeptide library Eur J Biochem 1996 240 1 71 77 10.1111/j.1432-1033.1996.0071h.x 8797837

[B38] Kropshofer H Max H Muller CA Hesse F Stevanovic S Jung G Kalbacher H Self-peptide released from class II HLA-DR1 exhibits a hydrophobic two-residue contact motif J Exp Med 1992 175 6 1799 1803 10.1084/jem.175.6.1799 1375272 PMC2119237

[B39] Smith KJ Pyrdol J Gauthier L Wiley DC Wucherpfennig KW Crystal structure of HLA-DR2 (DRA*0101, DRB1*1501) complexed with a peptide from human myelin basic protein J Exp Med 1998 188 8 1511 1520 10.1084/jem.188.8.1511 9782128 PMC2213406

[B40] Solache A Morgan CL Dodi AI Morte C Scott I Baboonian C Zal B Goldman J Grundy JE Madrigal JA Identification of three HLA-A*0201-restricted cytotoxic T cell epitopes in the cytomegalovirus protein pp65 that are conserved between eight strains of the virus J Immunol 1999 163 10 5512 5518 10553078

