# Supplementary material for: Prediction of peptides binding to MHC class I and II alleles by temporal motif mining
Source: BMC Bioinformatics. 2013 Jan 21;14(Suppl 2):S13. doi: 10.1186/1471-2105-14-S2-S13 (PMC3549809; doi:10.1186/1471-2105-14-S2-S13)
Supplement: Additional File 1 — The candidate and frequent itemsets of all lengths for the given example sequences in Figure 1, for minimum support value of 0.4. Red/bold values represent rules above the support threshold. At each step a candidate set Ck is generated by extending the last frequent itemset Lk-1, then the candidates are filtered according to the support values to generate the frequent itemset Lk. This process is repeated until no frequent itemsets of a certain size can be found. Afterwards, the resulting frequent sets of different sizes (except L1) are merged together and filtered according to a given minimum confidence boundary. [file 1471-2105-14-S2-S13-S1.docx]

| $C_{1}= \left\{ \begin{aligned} \boldsymbol{L, Supp:}\boldsymbol{7}/\boldsymbol{7} \\ \boldsymbol{V, Supp:}\boldsymbol{7}/\boldsymbol{7} \\ \boldsymbol{T, Supp:}\boldsymbol{6}/\boldsymbol{7} \\ \boldsymbol{G, Supp:}\boldsymbol{4}/\boldsymbol{7} \\ \boldsymbol{I, Sup}\boldsymbol{p:}\boldsymbol{4}/\boldsymbol{7} \\ \boldsymbol{A, Supp:}\boldsymbol{4}/\boldsymbol{7} \\ \boldsymbol{D, Supp:}\boldsymbol{3}/\boldsymbol{7} \\ Q, Supp:2/7 \\ N, Supp:2/7 \\ Y, Supp:2/7 \\ P, Supp:2/7 \\ E, Supp:1/7 \\ W, Supp:1/7 \\ K, Supp:1/7 \end{aligned} \right. {\to L}_{1}= \left\{ \begin{aligned} L, Supp:7/7 \\ V, Supp:7/7 \\ T, Supp:6/7 \\ G, Supp:4/7 \\ I, Supp:4/7 \\ A, Supp:4/7 \\ D, Supp:3/7 \end{aligned} \right.$ |
| --- |
| $C_{2}=L_{1}\to L_{1}= \left\{ \begin{aligned} \boldsymbol{L\to L, Supp:0.57} \\ \boldsymbol{L\to V, Supp:0.57} \\ \boldsymbol{L\to T, Supp:0.57} \\ \boldsymbol{L\to G, Supp:0.43} \\ L\to I, Supp:0.29 \\ \ldots\\ V\to L, Supp:0.29 \\ V\to V, Supp:0.29 \\ V\to T, Supp:0.29 \\ \boldsymbol{V\to G, Supp:0.57} \\ \ldots\end{aligned} \right. \to L_{2}=\left\{ \begin{aligned} L\to L, Supp: 0.57, Conf: 0.57 \\ L\to T, Supp: 0.57, Conf: 0.57 \\ L\to V, Supp: 0.57, Conf: 0.57 \\ V\to G, Supp: 0.57, Conf: 0.57 \\ T\to I, Supp: 0.43, Conf: 0.50 \\ T\to V, Supp: 0.43, Conf: 0.50 \\ I\to V, Supp: 0.43, Conf: 0.75 \\ L\to G, Supp: 0.43, Conf: 0.43 \\ L\to D, Supp: 0.43, Conf: 0.43 \end{aligned} \right.$ |
| $C_{3}=L_{2}\to L_{1}= \left\{ \begin{aligned} L-L\to L, Supp: 0.00 \\ \boldsymbol{L-L\to V, Supp:0.43} \\ L-L\to T, Supp:0.14 \\ \ldots\\ L-T\to L, Supp:0.00 \\ \boldsymbol{L-T\to V, Supp:0.43} \\ L-T\to T, Supp:0.14 \\ \ldots\end{aligned} \right. \to L_{3}= \left\{ \begin{aligned} L-L\to V, Supp:0.43, Conf:0.75 \\ L-T\to V, Supp:0.43, Conf:0.75 \end{aligned} \right.$ |
| $C_{4}=L_{3}\to L_{1}= \left\{ \begin{aligned} L-L-V\to L, Supp:0.00 \\ L-L-V\to V, Supp:0.00 \\ L-L-V\to T, Supp:0.14 \\ \ldots\\ L-T-V\to L, Supp:0.00 \\ L-T-V\to V, Supp:0.14 \\ L-T-V\to T, Supp:0.14 \\ \ldots\end{aligned} \right. \to L_{4}= Ø$ |
